# Mapping genetic determinants of host susceptibility to *Pseudomonas aeruginosa* lung infection in mice

**DOI:** 10.1186/s12864-016-2676-4

**Published:** 2016-05-11

**Authors:** Maura De Simone, Lorenza Spagnuolo, Nicola Ivan Lorè, Cristina Cigana, Ida De Fino, Karl W. Broman, Fuad A. Iraqi, Alessandra Bragonzi

**Affiliations:** Infection and Cystic Fibrosis Unit, IRCCS San Raffaele Scientific Institute, Milan, Italy; Department of Biostatistics & Medical Informatics, University of Wisconsin, Madison, WI USA; Department of Clinical Microbiology and Immunology, Sackler Faculty of Medicine, Tel Aviv University, Ramat Aviv, 69978 Tel Aviv, Israel

**Keywords:** *P. aeruginosa*, Pneumonia, Linkage analysis, Murine model, Host susceptibility, QTL mapping, Candidate genes

## Abstract

**Background:**

*P. aeruginosa* is one of the top three causes of opportunistic human bacterial infections. The remarkable variability in the clinical outcomes of this infection is thought to be associated with genetic predisposition. However, the genes underlying host susceptibility to *P. aeruginosa* infection are still largely unknown.

**Results:**

As a step towards mapping these genes, we applied a genome wide linkage analysis approach to a mouse model. A large F2 intercross population, obtained by mating *P. aeruginosa*-resistant C3H/HeOuJ, and susceptible A/J mice, was used for quantitative trait locus (QTL) mapping*.* The F2 progenies were challenged with a *P. aeruginosa* clinical strain and monitored for the survival time up to 7 days post-infection, as a disease phenotype associated trait. Selected phenotypic extremes of the F2 distribution were genotyped with high-density single nucleotide polymorphic (SNP) markers, and subsequently QTL analysis was performed. A significant locus was mapped on chromosome 6 and was named *P. aeruginosa infection resistance locus* 1 (*Pairl*1). The most promising candidate genes, including *Dok1, Tacr1, Cd207, Clec4f, Gp9, Gata2, Foxp1*, are related to pathogen sensing, neutrophils and macrophages recruitment and inflammatory processes.

**Conclusions:**

We propose a set of genes involved in the pathogenesis of *P. aeruginosa* infection that may be explored to complement human studies.

**Electronic supplementary material:**

The online version of this article (doi:10.1186/s12864-016-2676-4) contains supplementary material, which is available to authorized users.

## Background

*P. aeruginosa* is a versatile Gram-negative bacterium mainly implicated in pneumonia in intensive care settings and in patients with neutropenia, advanced AIDS, bronchiectasis, hereditary cystic fibrosis (CF) disease and advanced chronic obstructive pulmonary disease (COPD). Hospital-acquired pneumonia (HAP) occurs predominantly in patients with intubation and mechanical ventilation in an intensive care unit (ICU) [1, 2]. The risk of fatality in these patients is generally high but variable even with the correct use of antibiotics. Similarly to other infectious agents, exposure to *P. aeruginosa* results in a range of mild to severe clinical symptoms which can lead to fatal bacteremia in critically ill patients [3].

In general, the pathophysiology of human infections is a complex and dynamic process that depends on several factors: the genetic predisposition, the co-morbid conditions, the host immune response, the type of pathogen, and the site and extent of infection [4]. Most of the studies in the field of *P. aeruginosa* have been concentrated on the type of strains and the virulence factors [5–10]. A major contributing cause of *P. aeruginosa* predominance and pathogenesis was attributed to its ability to adapt to many different environments and cause a wide range of diseases in humans [11]. Less attention has been paid to host genetic predisposition as a contributing factor to the clinical outcome of *P. aeruginosa* infection. However, evidence of genetic predisposition is emerging in the CF and COPD populations. In CF, it has been shown that the progression and severity of pulmonary disease do not appear to correlate exclusively with specific mutations in the causative gene cystic fibrosis transmembrane conductance regulator (*CFTR*) but appear to be largely dependent on other modifier genes [12, 13]. In COPD, several host candidate genes have been proposed as causing the acceleration of lung function decline but have never been associated with *P. aeruginosa* infection [14, 15]. Regarding other patients at risk of *P. aeruginosa* infection, no study has yet been reported. The size of cohorts, strong but often unknown environmental influences, poor diagnosis, and lack of repeatability limit scientific advancements in understanding the genetic basis of susceptibility to *P. aeruginosa* diseases in humans.

Inbred mice, with defined and reproducible genetics, can be maintained in standardized environments and exposed to identical challenges, unlike human populations. These aspects allow us to directly correlate the phenotypic response to the specific genotype. Inbred mice have been successfully adopted to allow us to understand the genetic basis of differential susceptibility to infections (e.g. bacteria, viruses and parasites) [16, 17]. When genes are identified in a mouse model, human orthologue genes can subsequently be identified. In the context of *P. aeruginosa* infection, variability in survival, magnitude of inflammatory response, bacterial clearance and lung damage have been reported in various inbred strains [18–22]. Recently, we also showed that the genetic background of the host influences the response to *P. aeruginosa* infection [23]. These data were obtained by ranking nine inbred murine strains characterized by a wealth of genetic and phenotypic diversity. In particular, A/J and C3H/HeOuJ showed the most deviant clinical (e.g. body weight and mortality) and immunological phenotypes (e.g. cytokines and innate immune cells response). These results provided a basis for mapping genomic regions underlying host susceptibility to *P. aeruginosa* infection. Among different traits recorded, mortality caused by bacteremia was considered as the ultimate clinical expression of the deleterious clash between the host immune response and invasive microorganisms. Consequently, in this study we generated an informative F2 population by crossing between the parental strains, including resistant C3H/HeOuJ and susceptible A/J mice and subsequently performed quantitative trait loci (QTLs) mapping analysis. We identified a genetic locus on murine chromosome 6, designated *P. aeruginosa infection resistance locus* 1 (*Pairl*1), that has major effect on the susceptibility to *P. aeruginosa* infection and risk of bacteremia.

## Methods

### Ethic statement

Animal studies were conducted according to protocols approved by San Raffaele Scientific Institute (Milan, Italy) Institutional Animal Care and Use Committee (IACUC) and adhered strictly to the Italian Ministry of Health guidelines for the use and care of experimental animals (Permit number: 502). Research on the bacterial isolates AA2, from the individual with CF, used for animal experiments, has been approved by the Ethics Commission of Hannover Medical School, Germany. The patients and parents gave informed verbal consent before the sample collection. Approval for storing of biological materials was obtained by the Ethics Commission of Hannover Medical School, Germany.

### Bacterial strain

*P. aeruginosa* clinical isolate AA2 was described before [6, 23, 24]. The strain was cultured in trypticase soy broth (TSB) and plated on trypticase soy agar (TSA).

### Mice, breeding and *P. aeruginosa* lung infection

Based on our previous study, we have determined that A/J and C3H/HeOuJ are susceptible and resistant to *P. aeruginosa* infection [23], respectively 7–8 weeks old male and female mice of A/J and C3H/HeOuJ were purchased from Jackson Laboratory, and used as the parental founders of F2 mapping population. An outcross between the two founders was conducted and subsequently the members of the first generation (F1 population) were intercrossed to generate 400 (A/J X C3H/HeOuJ) F2 mice. A/J, (*n* = 22) C3H/HeOuJ (*n* = 26), and (A/J x C3H/HeOuJ) F2 mice (*n* = 400) were intratracheal injected with a dose of 5 × 10^6^ of the planktonic *P. aeruginosa* clinical isolate AA2, and monitored for survival up to 7 days according with established procedures [23].

Mice were maintained in pathogen free conditions during breeding and transferred in biosafety level 3 stabulary for infection experiments. Mice were observed daily for clinical signs including coat quality, posture, ambulation, and hydration status.

Statistical significance by Mantel-Cox test was performed to compare the survival between pairs. The data are pooled at least from three independent experiments.

### Genotyping

We applied a selective genotyping approach, as suggested by Darvasi and Soller [25], whose power was successfully proven and confirmed in several previous studies [26–30]. We genotyped only the phenotypic extremes of the entire F2 outcross population. A total of 160 mice of the F2 population (20 % of highly and 20 % lowly susceptible) were genotyped and genotypes were randomized by meiosis.

High molecular genomic DNA samples of the 160 F2 mice and the parental mouse strains were extracted from tail biopsies with the DNeasy Blood and Tissue (Qiagen, Hilden, Germany). Samples were analyzed for purity (A_260_/A_280_) and DNA content was evaluated by NanoDrop measurement. The genomic DNA samples of the F2 progeny were genotyped with MegaMUGA mouse array which consists 77,000 SNP markers based on the Illumina® Infinium platform [31]. The SNP genotype was conducted at Neogen company (Neogen/Geneseek, Lincoln, NE, USA). The SNP markers are distributed across the entire genome with an average spacing of 33 Kb.

### QTL analysis

To map QTL contributing to survival, we used Haley-Knott regression [32], including sex as an additive covariate (that is, allowing for a shift in average survival between the two sexes, but assuming that the effect of a QTL is the same in both sexes). Statistical significance was assessed by a permutation test [33], correcting for the multiple statistical tests inherent in the scan of the genome. Sample size was chosen to give 80 % power to detect a QTL explaining ~5 % of the phenotypic variance. A confidence interval for the location of the inferred QTL was derived as a 1.5-LOD support interval [34]. Calculations were performed with R/qtl [35], an add-on package to the general statistical software R (R Development Core Team 2015).

### Candidate genes analysis

The public database Mouse Genome Informatics (MGI, http://www.informatics.jax.org/) was used for the identification of suggested genes within the QTL interval and for prioritization together with Endeavour [36, 37]. To generate the training sets of genes employed for the analysis, we used the ‘key word’ approach described previously [38, 39].

### Statistical analysis

We used Mantel-Cox test to compare the survival between pairs. Tests were considered statistically significant when the significance level was ≤ 0.05.

## Results

### Susceptibility to *P. aeruginosa* pulmonary infection in (A/JxC3H/HeOuJ) F2 progenies

**Fig. 1:**
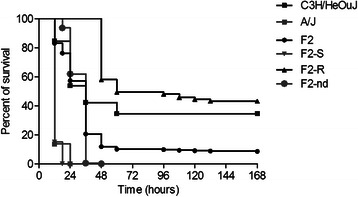
Survival curves of A/J, C3H/HeOuJ and (AJ x C3H/HeOuJ) F2 population after acute *P. aeruginosa* lung infection. A/J (*n* = 22), C3H/HeOuJ (*n* = 26), and (A/J x C3H/HeOuJ) F2 mice (F2 complete population *n* = 400; F2-S: 20 % most susceptible genotyped, *n* = 80; F2-R: 20 % most resistant genotyped, *n* = 80; F2-nd not detected genotype, *n* = 240) were intratracheal injected with a dose of 5 × 10^6^ of the planktonic *P. aeruginosa* clinical isolate AA2, and monitored for survival up to 168 h (7 days). Statistical significance by Mantel-Cox test is reported in Additional file 1

**Fig. 2:**
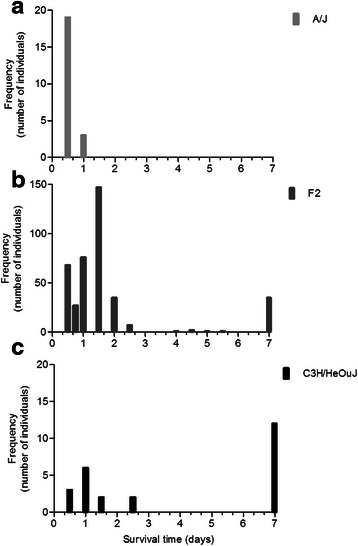
Histograms of survival time of A/J, C3H/HeOuJ and (AJxC3H/HeOuJ) F2 population after acute *P. aeruginosa* lung infection. **a** A/J (*n* = 22), **b** C3H/HeOuJ (*n* = 26), and **c** (A/J x C3H/HeOuJ) F2 mice (*n* = 400) were intratracheal injected with a dose of 5 × 10^6^ of the planktonic *P. aeruginosa* clinical isolate AA2, and monitored for survival up to 7 days

Our experiments established that the intratracheal injection of 5 × 10^6^ CFU of the *P. aeruginosa* clinical isolate AA2 causes the death of A/J mice within 24 h, whereas a set of C3H/HeOuJ mice (~40 %) survives for more than 7 days (Fig. 1). The selected A/J and C3H/HeOuJ parental inbred mouse strains were highly and significantly divergent in the risk of sepsis (*p* < 0.0001, Mantel-Cox test). To analyse the potential contribution of genetic factors to *P. aeruginosa* infection, the F1 population and subsequently 400 (A/JxC3H/HeOuJ) F2 mice were generated by reciprocal mating of susceptible A/J and resistant C3H/HeOuJ mice. The entire F2 population was infected with *P. aeruginosa* as described above and survival of each mouse was recorded over a period of 7 days post-infection. F2 progenies showed a survival which was significantly higher than the susceptible strain A/J, but lower when compared to C3H/HeOuJ parental lines (Fig. 1). Then, to collect data for the subsequent genome analysis, the F2 population was sorted based on the phenotypic extremes - the most susceptible (F2-S) (20 % of F2 population *n* = 80) and most resistant (F2-R) (20 % of F2 population *n* = 80) -. The selected F2-S showed a significantly lower survival compared to C3H/HeOuJ mice (Hazard Ratio 0.03057, 95 % CI 0.01386 to 0.06741; *p* < 0.0001, Mantel-Cox test) but similar to A/J mice (Hazard Ratio 0.4345, 95 % CI 0.1288 to 1.466) while F2-R showed a significantly higher survival compared to the parental C3H/HeOuJ (Hazard Ratio 2.969, 95 % CI 1.382 to 6.378; *p* = 0.0053, Mantel-Cox test) and A/J mice (Hazard Ratio 688.9, 95 % CI210.3 to 2256; *p* < 0.0001,Mantel-Cox test). The rest of the F2 population (Fig. 1 as not detected genotype, F2-nd) showed survival kinetics that were significantly different from F2-S and F2 -R (Hazard ratio 0.004038, 95 % CI 0.002248 to 0.007256 for F2-S, and Hazard ratio 18.56,95 % CI 12.65 to 27.21 for F2-R; *p* < 0.0001, Mantel-Cox test) and the parental lines (Hazard Ratio 0.3129, 95 % CI 0.1486 to 0.6589 for C3H/HeOuJ; *p* = 0.0022 Mantel-Cox test, and Hazard Ratio 5785, 95 % CI 1549 to 21606 for A/J;*p* < 0.0001, Mantel-Cox test). Finally the entire F2 population (*n* = 400) showed a significantly higher survival compared to the parental A/J (Hazard Ratio 49.79, 95 % CI 19.16 to 129.4;*p* < 0.0001, Mantel- Cox test) a slightly significantly lower survival when compared to the parental C3H/HeOuJ (Hazard Ratio 0.5007, 95 % CI 0.3270 to 0.8615; *p* = 0.0104 Mantel- Cox test). The complete statistical analysis is reported in Additional file 1 (Table S1). The distribution histogram of the survival time in the F2 population, with values ranging from 0,5 to 7 days, is shown in Fig. 2b, along with the distribution in A/J mice (Fig. 2a) and C3H/HeOuJ mice (Fig. 2c).

Additional analysis showed that high numbers of CFUs (average of 1 × 10^6^ in the whole spleen) were present in the moribund mice, both parental and their progenies, indicating sepsis as the cause of death (data not shown).

### Mapping the genetic determinants to *P. aeruginosa* susceptibility

**Fig. 3:**
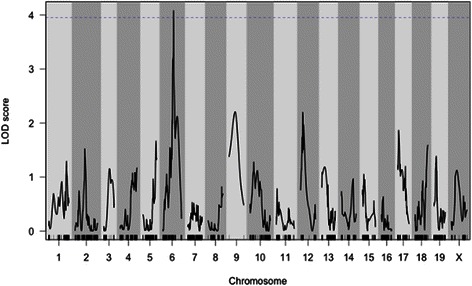
QTL scan showing LOD score (Y-axis) and genome position (X-axis) for susceptibility to *P. aeruginosa* infection. The x axis shows chromosome numbers, the y axis shows the statistical significance of the association measured as LOD, logarithm of the odds favouring linkage, a score that measures the strength of evidence for the presence of a QTL among the 160 mice of the F2 population, including F2-S and F2-R. The horizontal line is plotted at the 5 % significance threshold. One significant QTL (*p* = 0.037) named *Pairl 1* was mapped on chromosome 6 at 90.8 Mbp with 4.3 LOD score, with a 1.5-LOD drop support interval of 81.5–102.2 Mbp

In the next phase, we proceeded to map the genetic factors that contributed to susceptibility to the *P. aeruginosa* acute lung infection. The survival time was used as the main phenotypic trait for mapping QTL associated with host susceptibility to this opportunistic pathogen. In order to maximize the ability to detect the QTLs, we took advantage of the selective genotyping approach, described previously [25] and successfully applied in several studies [27–29]. The phenotypic extremes F2-S and F2-R were genotyped with about 10 K informative SNP markers selected from MegaMuga SNP array, and subsequently QTL mapping was performed. We identified a significant QTL on chromosome 6 (*p* = 0.037) at map position 90.8 Mbp with LOD Score = 4.3, and with a 1.5-LOD support genomic interval of 81.5–102.2 Mbp (Fig. 3). The SNPs markers used to genotype the locus of interest on chromosome 6 are reported in Additional file 2 (Table S2). We defined the mapped QTL on chromosome 6 as *Pairl*1 for *P. aeruginosa infection resistance locus* 1.

The effect of the locus on survival is presented in Additional file 3 (Figure S1). The locus shows some degree of over-dominance, with the heterozygotes surviving longer than the homozygotes (A/J homozygotes versus heterozygotes *p* = 0.0056 Mantel-Cox test; C3H/HeOuJ homozygotes versus heterozygotes *p* < 0.0001, Mantel-Cox test) and a transgressive allele segregation with the A/J homozygotes surviving longer than the C3H/HeOuJ homozygotes. This is the opposite what would be expected from the parental strains’ phenotypes.

### Identification and functional classification of candidate genes

A total of 323 genes within the *Pairl1* interval were identified from the public database (http://informatics.jax.org/), as described in Material and methods and classified as protein coding genes (*n* = 198), RNA genes (*n* = 50), unclassified genes (*n* = 45) and unclassified non-coding RNA genes (*n* = 30). The full list of genes in the interval is reported in Additional file 4 (Table S3). Gene prioritization was: i) carried out by integrative computational analysis of public and private genomic data [38], ii) based on training lists of genes involved in relevant biological and pathological processes and iii) ranked based on literature research.

To prioritize our genes, we used Endeavour and the MGI database (http://informatics.jax.org/). For Endeavour, candidates genes are first ranked according to their similarity to a list of known disease training genes involved in lung infection, bacteremia, pneumonia, sepsis, chemotaxis, *P. aeruginosa*, innate immune response, pattern recognition receptor, neutrophils, complement, CF, modifier genes in CF, and COPD (Additional file 5A: Table S4A). For the MGI database, we based the search on different keywords (Lung, Infection, Respiratory AND Infection) and gene ontology terms (Immune AND Response, Cytokines, Chemotaxis) (Additional file 5B: Table S4B). Interestingly, the two different approaches revealed several redundant genes and consequently, we proceeded with a systematic literature research to confirm a possible role in susceptibility to *P. aeruginosa* infection. The candidate genes in Table 1 are those revealed by the literature research as having a putative role in host defense to infection. In particular, the most promising candidate genes (*Dok1, Tacr1, Cd207, Clec4f, Gp9, Gata2, Foxp1*) were those involved in pathogen sensing, neutrophils and macrophages recruitment, and inflammatory process. Our prioritization of results depends on the adopted criteria for the bioinformatics analysis and on the bioinformatics tool itself. Therefore, it cannot be excluded that other possible candidate genes present in our list could somehow play a role in *P. aeruginosa* infection.

**Table 1:** The list of the most promising candidate genes identified in the *Pairl1* locus

Gene name	Biological function	References
*Dok1*	Adaptor protein involved in: • lung homeostasis • negative regulation of LPS signaling • Downregulation of its expression in in vitro models	[28, 29]
*Tacr1*	Receptor for neuropeptide tachykinin involved in: • Cytokines and chemokines expression • Chemotaxis and neutrophils activation • *P. aeruginosa* corneal infection	[30–32]
*Cd207* *Clecf4*	Members of C-type lectin superfamily involved in: • Pathogen sensing of virus, fungi and bacteria	[33–35]
*Gp9*	Glycoprotein 9-Glycoprotein Ib complex involved in: • Leukocytes migration • Inflammation • Neutrophils extracellular trap formation	[36]
*Foxp1* *Gata2*	Transcriptional factors involved in: • Cytokines expression	[37, 38]

## Discussion

Phenotypic variations of host response to *P. aeruginosa* infection have been demonstrated in several inbred mouse strains [18–22] and in the most recent Collaborative Cross population [40 ]. In our earlier work, we showed that classical inbred strains of mice manifest an extreme response - highly resistant or highly susceptible - to *P. aeruginosa* infection [23]. In particular, the risk of fatality in A/J mice was high after exposure to *P. aeruginosa* while C3H/HeOuJ showed milder clinical symptoms associated to higher resolution of the infection. However, the genetic component of this trait is still poorly understood. For the first time to our knowledge, in this study, we localized a genetic determinant of susceptibility to *P. aeruginosa* respiratory infection. First, we generated an informative F2 population by crossing the resistant C3H/HeOuJ and susceptible A/J mice. Next, we scored this F2 population for survival time, as the main associated disease phenotypic trait, and subsequently used it for QTL mapping. The *P. aeruginosa* dose for infection of the newly generated populations was selected according to the most deviant differences in survival between parental lines. The F2 population showed a wide range of responses in terms of survival compared to the parental lines. For this study, we used *P. aeruginosa* isolate sampled at the onset of infection from a CF patient and fully characterized in previous studies [41–44 ]. Although the *P. aeruginosa* strain was selected on the basis of its similarity to environmental strains, including genotypic and phenotypic features, it is possible that this particular strain may affect several physiological parameters and subsequent analysis.

Our genetic linkage approach was successful in identifying for the first time the *Pairl1* locus, on murine chromosome 6, linked to susceptibility to *P. aeruginosa* pulmonary infection with a genome wide significance of 95 % (*p* < 0.05). Here, the gene prioritization approach was used to rank genes involved in the susceptibility to *P. aeruginosa* infection. This approach was applied successfully in candidate gene analysis in other diseases such as obesity, Type 2 diabetes, and asthma [45, 46 ]. The most promising candidate genes included in the *Pairl1* locus, namely *Dok1, Tacr1, Cd207, Clec4f, Gp9, Gata2* and *Foxp1*, are mainly involved in pathogen sensing, neutrophils recruitment and cytokines response.

In more detail, as *Dok1 (Docking protein1)* belongs to a family of adaptor proteins, it plays a critical anti-inflammatory role in lung homeostasis. It is involved in negative regulation of LPS signaling and its expression is downregulated after *P. aeruginosa* infection in vitro [47, 48]. Together with Dok 2 and Dok 3, it plays essential roles in negative regulation of a wide variety of signaling pathways in both innate and adaptive immunities [49]. Mice knock-out for Dok1 were hypersensitive to LPS showing high levels of TNFα and NO [48] while triple KO mice (Dok1-2-3) showed spontaneous pulmonary inflammation, with hallmarks of asthma, including eosinophilia, goblet cell hyperplasia, and subepithelial fibrosis [50]. Most importantly, downregulation of *Dok1* gene expression in human alveolar macrophages after *P. aeruginosa* infection in vitro, indicates its direct involvement in this bacterial infection [48].

The gene *Tacr1 (Tachykinin receptor 1)* encodes the receptor for the neuropeptide tachykinin and is implicated in mediating a variety of proinflammatory processes, including upregulation of cytokine and chemokine expression, chemotaxis and activation of inflammatory cells, all pathways that seem to be altered in susceptible A/J mice during acute *P. aeruginosa* infection [51]. Moreover, the binding of this receptor with its ligand has been shown to increase the neutrophils adhesion to bronchial epithelial cells [52]. More interestingly, *Tacr1* has been implicated in several bacterial infections and in sepsis [53, 54], including *P. aeruginosa* corneal infection [55].

*Cd207* (*C-type lectin domain family 4 member* K) gene encodes a type II transmembrane, C-type lectin receptor displaying mannose-binding specificity. It has high affinity for mannose structures abundantly expressed by pathogens, especially by viruses (HIV-1 and HSV) as well as the mycobacterial cell wall component mannosylated lipoarabinomannan (ManLAM) and pathogenic funguses such as *Candida species, Malassezia furfur* and *Saccharomyces cerevisiae* [56]. The gene *Clec4f* shows high similarity with *Cd207,* and therefore might be involved in the same processes. The importance of these genes in the context of *P. aeruginosa* infection might be seen in the functional association of this C-type lectin receptor with the Mannose Binding Lectin 2 (MBL2) gene, which codes for the MBL, a well-known modifier gene in CF. In fact, polymorphisms in MBL2 are associated with differing level of MBL production, and deficiencies in MBL production have been linked to an increased incidence of infection, [57]. In particular it predisposes to early infection with *P. aeruginosa* which in turn, leads to more severe lung disease [58].

Interestingly, other candidates in the *Pairl1* locus play important roles in leukocytes recruitment. Specifically, *Gp9 (glycoprotein 9),* expressed by platelets, is involved in leukocyte migration, inflammation and promotion of neutrophil extracellular trap formation [59]. In a mouse model of sepsis this glycoprotein complex contributes to a platelet/neutrophil and platelet/monocyte axis with significant consequences to the innate immune response in terms of elevated serum cytokine levels and Mac1 increased expression by neutrophils for their extracellular cell migration. Among the candidate genes, we also identified the transcription factors *Foxp1 (Forkhead box P1)* and *Gata2 (GATA binding protein 2)* that are involved in pro-inflammatory cytokines expression, such as IL1ß, IL-6 and IL-17RA correlated to the Th17 pathway (http://www.innatedb.com/) [60].

## Conclusions

We suggest promising candidates for the genetic basis of host susceptibility to *P. aeruginosa* infection. In addition to a better understanding of host pathogen interactions, the characterization of these genes may have significant implications for the discovery of novel possible therapeutic targets and/or prognostic biomarkers complementing human studies.

## Ethics

Animal studies were conducted according to protocols approved by San Raffaele Scientific Institute (Milan, Italy) Institutional Animal Care and Use Committee (IACUC) and adhered strictly to the Italian Ministry of Health guidelines for the use and care of experimental animals (Permit number: 502). Research on the bacterial isolates AA2, from the individual with CF, used for animal experiments, has been approved by the Ethics Commission of Hannover Medical School, Germany. The patients and parents gave informed verbal consent before the sample collection. Approval for storing of biological materials was obtained by the Ethics Commission of Hannover Medical School, Germany.

## Consent to publish

Not applicable.

## Availability of data and materials

The data sets supporting the results of this article are included within the article and its additional files.

## Additional files

Additional file 1: Statistical comparison of survival time between A/J, C3H/HeOuJ and (AJxC3H/HeOuJ) F2 population after acute *P. aeruginosa* lung infection. (DOCX 15 kb) Additional file 2: Recombination map position of 38 SNPs markers in the locus of interest of F2 Chromosome 6. (DOCX 16 kb) Additional file 3: Effect of the *Pairl*1 locus on survival in A/J, C3H/HeOuJ and heterozygotes. (DOCX 106 kb) Additional file 4: List of all the genes within the *Pairl1* locus obtained from public database Mouse Genome Informatics (MGI, http://www.informatic.jax.org). (DOCX 66 kb) Additional file 5: List of the most promising candidates genes for susceptibility to acute *P. aeruginosa* pulmonary infection. (DOCX 27 kb)

## Abbreviations

CF: cystic fibrosis
CFTR: cystic fibrosis transmembrane conductance regulator
COPD: chronic obstructive pulmonary disease
Cd207 and Clecf4: C-type lectin domain family 4 member f and K
Dok1: Docking protein1
Foxp1: Forkhead box P1
Gata2: GATA binding protein 2
Gp9: glycoprotein 9
ICU: intensive care unit
QTL: quantitative trait locus
Tacr1: Tachykinin receptor 1
