# Supplementary material for: Mapping genetic determinants of host susceptibility to Pseudomonas aeruginosa lung infection in mice
Source: BMC Genomics. 2016 May 11;17:351. doi: 10.1186/s12864-016-2676-4 (PMC4866434; doi:10.1186/s12864-016-2676-4)
Supplement: Additional file 1: — Statistical comparison of survival time between A/J, C3H/HeOuJ and (AJxC3H/HeOuJ) F2 population after acute P. aeruginosa lung infection. (DOCX 15 kb) [file 12864_2016_2676_MOESM1_ESM.docx]

**Table S1. Statistical comparison of survival time between A/J, C3H/HeOuJ and (AJxC3H/HeOuJ) F2 population after acute *P. aeruginosa* lung infection*.***

| **Strain** | **C3H/HeOuJ** | | **F2** | **F2-S** | **F2-R** | **F2-nd** |
| --- | --- | --- | --- | --- | --- | --- |
| **A/J** | **** | **** | | ns | **** | **** |
| **C3H/HeOuJ** | ns | *(0.0104) | | **** | **(0.0053) | **(0.0022) |
| **F2** | *(0.0104) | ns | | ns | ns | ns |
| **F2-S** | **** | **** | | ns | **** | **** |
| **F2-R** | **** | **** | | **** | ns | **** |
| **F2-nd** | **(0.0022) | ns | | **** | **** | ns |
|  |  |  | |  |  |  |

Statistical significance by Mantel-Cox test was used to compare survival between pairs and is indicated :* p<0.05 , **p<0.01,****p<0.0001.
