# Supplementary material for: Mapping genetic determinants of host susceptibility to Pseudomonas aeruginosa lung infection in mice
Source: BMC Genomics. 2016 May 11;17:351. doi: 10.1186/s12864-016-2676-4 (PMC4866434; doi:10.1186/s12864-016-2676-4)
Supplement: Additional file 2: — Recombination map position of 38 SNPs markers in the locus of interest of F2 Chromosome 6. (DOCX 16 kb) [file 12864_2016_2676_MOESM2_ESM.docx]

**Table S2**: **Recombination map position of 38 SNPs markers in the locus of interest of F2 Chromosome 6**

CentiMorgan (cM) positions of 38 SNPs markers are reported based on The Jackson Laboratory’s online Mouse Genome Informatics resource (MGI, <http://www.informatic.jax.org>)

| Chromosome 6 marker | MGI recombinant map position (cM) |
| --- | --- |
| UNC11461982 | 38.86 |
| UNC11464537 | 38.86 |
| UNC11466512 | 38.86 |
| UNC11470537 | 38.86 |
| UNC11473457 | 38.66 |
| UNC11474388 | 38.86 |
| UNC11478679 | 39.49 |
| UNC11479717 | 39.49 |
| UNC11482526 | 39.49 |
| UNC11487129 | 39.81 |
| UNC11493715 | 39.81 |
| UNC11497244 | 39.81 |
| UNC11500109 | 39.81 |
| UNC060133816 | 39.81 |
| UNC11502976 | 39.81 |
| UNC11503636 | 39.81 |
| UNC11504615 | 39.81 |
| UNC11507130 | 39.81 |
| UNC060135143 | 40.12 |
| UNC11517362 | 40.12 |
| UNC11518055 | 40.12 |
| UNC11518626 | 40.12 |
| UNC060135275 | 40.44 |
| UNC060387481 | 40.77 |
| UNC11520628 | 40.77 |
| UNC11555406 | 42.35 |
| UNC11555784 | 42.35 |
| UNC060388048 | 42.35 |
| UNC11557049 | 42.35 |
| UNC11557076 | 42.35 |
| UNC11555784 | 42.35 |
| UNC11564421 | 42.35 |
| UNC11577366 | 43.29 |
| UNC11564970 | 42.35 |
| UNC11566037 | 42.35 |
| UNC11566754 | 42.35 |
| UNC11567068 | 42.35 |
| UNC11567464 | 42.35 |
