# Supplementary material for: Mapping genetic determinants of host susceptibility to Pseudomonas aeruginosa lung infection in mice
Source: BMC Genomics. 2016 May 11;17:351. doi: 10.1186/s12864-016-2676-4 (PMC4866434; doi:10.1186/s12864-016-2676-4)
Supplement: Additional file 3: — Effect of the Pairl1 locus on survival in A/J, C3H/HeOuJ and heterozygotes. (DOCX 106 kb) [file 12864_2016_2676_MOESM3_ESM.docx]

**Figure S1. Effect of the *Pairl*1 locus on survival in A/J, C3H/HeOuJ homozygotes and heterozygotes.**

Effects of the *Pairl*1 locus, based on percent of survival, with the three groups of mice based on its genotypes at the nearest maker (UNC11577366) to the QTL peak on Chromosome 6 (**A**), and the average for each group was plotted vs. genotype where A and C represent A/J and C3H/HeOuJ genotype respectively (**B**). The locus shows some degree of over-dominance, with the heterozygotes surviving longer than parental lines and a transgressive allele segregation with the A/J homozygotes surviving longer than the C3H/HeOuJ homozygotes. This is the opposite what would be expected from the parental strains’ phenotypes (shown in Figure 1). Statistical significance by Mantel-Cox test was used to compare survival between pairs and is indicated :** p<0.01 ,****p<0.0001

**
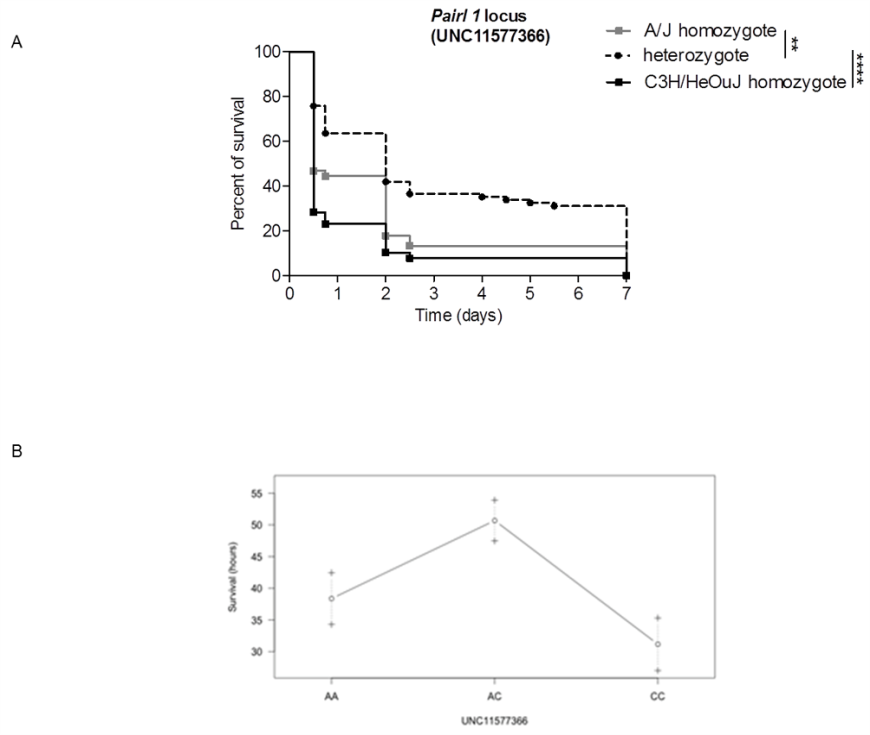
**
