# Supplementary material for: Mapping genetic determinants of host susceptibility to Pseudomonas aeruginosa lung infection in mice
Source: BMC Genomics. 2016 May 11;17:351. doi: 10.1186/s12864-016-2676-4 (PMC4866434; doi:10.1186/s12864-016-2676-4)
Supplement: Additional file 4: — List of all the genes within the Pairl1 locus obtained from public database Mouse Genome Informatics (MGI, http://www.informatic.jax.org). (DOCX 66 kb) [file 12864_2016_2676_MOESM4_ESM.docx]

**Table S3. List of all the genes within the *Pairl1* locus obtained from public database Mouse Genome Informatics (MGI,** <http://www.informatic.jax.org>)

| Chromosome | Start | End | cM | strand GRCm38 | MGI ID | Feature Type | Symbol | Name |
| --- | --- | --- | --- | --- | --- | --- | --- | --- |
| 6 | 50864934 | 145656013 |  |  | MGI:3032560 | heritable phenotypic marker | Sndy1 | syndactyly 1 |
|  |  |  |  |  |  |  |  |  |
| 6 | 78629504 | 92040033 |  |  | MGI:3771720 | heritable phenotypic marker | wa1l | waved 1-like |
| 6 | 81638311 | 81638469 |  | + | MGI:5456041 | snRNA gene | Gm26264 | predicted gene, 26264 |
| 6 | 81806791 | 81806908 |  | + | MGI:5452307 | snoRNA gene | Gm22530 | predicted gene, 22530 |
| 6 | 81893820 | 81895338 |  |  | MGI:1920811 | unclassified gene | 1700097M23Rik | RIKEN cDNA 1700097M23 gene |
| 6 | 81895590 | 81895663 |  | - | MGI:5562768 | miRNA gene | Mir7232 | microRNA 7232 |
| 6 | 81896599 | 81896676 | 35.81 | - | MGI:3619414 | miRNA gene | Mir468 | microRNA 468 |
| 6 | 81900464 | 81910800 |  | - | MGI:1921470 | unclassified non-coding RNA gene | 1700009C05Rik | RIKEN cDNA 1700009C05 gene |
| 6 | 81923669 | 81959915 | 35.81 | + | MGI:2141656 | protein coding gene | Gcfc2 | GC-rich sequence DNA binding factor 2 |
| 6 | 81957851 | 81965958 | 35.81 | - | MGI:1926274 | protein coding gene | Mrpl19 | mitochondrial ribosomal protein L19 |
| 6 | 81984321 | 81992426 | 35.81 | + | MGI:5012568 | unclassified non-coding RNA gene | Gm20383 | predicted gene, 20383 |
| 6 | 82041043 | 82093099 | 35.81 | + | MGI:2385247 | protein coding gene | Eva1a | eva-1 homolog A (C. elegans) |
| 6 | 82050453 | 82052581 | 35.81 | - | MGI:3802115 | unclassified gene | Gm15864 | predicted gene 15864 |
| 6 | 82402475 | 82560103 | 35.94 | + | MGI:98475 | protein coding gene | Tacr1 | tachykinin receptor 1 |
| 6 | 82618992 | 82705365 | 35.94 | - | MGI:1914229 | protein coding gene | Pole4 | polymerase (DNA-directed), epsilon 4 (p12 subunit) |
| 6 | 82725025 | 82774454 | 35.94 | - | MGI:1315197 | protein coding gene | Hk2 | hexokinase 2 |
| 6 | 82764282 | 82767288 | 35.94 | + | MGI:4937861 | unclassified non-coding RNA gene | Gm17034 | predicted gene 17034 |
| 6 | 82877864 | 82881853 |  | - | MGI:1916902 | unclassified non-coding RNA gene | 2310069B03Rik | RIKEN cDNA 2310069B03 gene |
| 6 | 82911885 | 82939769 | 35.94 | - | MGI:1340055 | protein coding gene | Sema4f | sema domain, immunoglobulin domain (Ig), TM domain, and short cytoplasmic domain |
| 6 | 82946902 | 83030183 | 35.94 | + | MGI:1315200 | protein coding gene | M1ap | meiosis 1 associated protein |
| 6 | 83030934 | 83033471 | 35.94 | - | MGI:893587 | protein coding gene | Dok1 | docking protein 1 |
| 6 | 83034173 | 83052562 | 35.94 | + | MGI:1337004 | protein coding gene | Loxl3 | lysyl oxidase-like 3 |
| 6 | 83049711 | 83049773 |  | + | MGI:5530871 | miRNA gene | Mir7040 | microRNA 7040 |
| 6 | 83051266 | 83055273 | 35.94 | - | MGI:1928676 | protein coding gene | Htra2 | HtrA serine peptidase 2 |
| 6 | 83054653 | 83057680 | 35.94 | + | MGI:107789 | protein coding gene | Aup1 | ancient ubiquitous protein 1 |
| 6 | 83057844 | 83067210 | 35.94 | + | MGI:2136388 | protein coding gene | Dqx1 | DEAQ RNA-dependent ATPase |
| 6 | 83068324 | 83070225 | 35.94 | - | MGI:1350935 | protein coding gene | Tlx2 | T cell leukemia, homeobox 2 |
| 6 | 83077869 | 83080855 | 35.94 | + | MGI:1917087 | protein coding gene | Pcgf1 | polycomb group ring finger 1 |
| 6 | 83086367 | 83088243 | 35.94 | + | MGI:1342288 | protein coding gene | Lbx2 | ladybird homeobox homolog 2 (Drosophila) |
| 6 | 83090311 | 83090387 | 35.94 | - | MGI:4441434 | miRNA gene | Mir3470a | microRNA 3470a |
| 6 | 83101617 | 83109939 | 35.94 | + | MGI:3045292 | protein coding gene | Ccdc142 | coiled-coil domain containing 142 |
| 6 | 83106608 | 83109054 | 35.94 | - | MGI:3783052 | unclassified gene | Ccdc142os | coiled-coil domain containing 142, opposite strand |
| 6 | 83109095 | 83109939 | 35.94 | + | MGI:1915749 | protein coding gene | Mrpl53 | mitochondrial ribosomal protein L53 |
| 6 | 83115496 | 83118898 | 35.94 | + | MGI:1929872 | protein coding gene | Mogs | mannosyl-oligosaccharide glucosidase |
| 6 | 83119044 | 83121559 | 35.94 | - | MGI:104710 | protein coding gene | Wbp1 | WW domain binding protein 1 |
| 6 | 83121765 | 83125431 | 35.94 | - | MGI:1917270 | protein coding gene | Ino80b | INO80 complex subunit B |
| 6 | 83135463 | 83152579 | 35.94 | + | MGI:107371 | protein coding gene | Rtkn | rhotekin |
| 6 | 83149361 | 83156397 | 35.94 | - | MGI:1922909 | protein coding gene | Wdr54 | WD repeat domain 54 |
| 6 | 83156404 | 83162975 | 35.94 | + | MGI:1919087 | protein coding gene | 1700003E16Rik | RIKEN cDNA 1700003E16 gene |
| 6 | 83165920 | 83200117 | 35.94 | + | MGI:107745 | protein coding gene | Dctn1 | dynactin 1 |
| 6 | 83166152 | 83172733 | 35.94 | - | MGI:3783068 | unclassified gene | Gm15624 | predicted gene 15624 |
| 6 | 83219828 | 83304945 | 35.94 | + | MGI:2443220 | protein coding gene | Slc4a5 | solute carrier family 4, sodium bicarbonate cotransporter, member 5 |
| 6 | 83305691 | 83317606 | 35.94 | - | MGI:1338850 | protein coding gene | Mthfd2 | methylenetetrahydrofolate dehydrogenase (NAD+ dependent), methenyltetrahydrofolate cyclohydrolase |
| 6 | 83326016 | 83343776 | 35.94 | + | MGI:2442631 | protein coding gene | Mob1a | MOB kinase activator 1A |
| 6 | 83349147 | 83360136 | 35.94 | + | MGI:1925903 | protein coding gene | Bola3 | bolA-like 3 (E. coli) |
| 6 | 83362373 | 83459084 | 35.94 | - | MGI:2446229 | protein coding gene | Tet3 | tet methylcytosine dioxygenase 3 |
| 6 | 83400996 | 83401102 |  | - | MGI:5530674 | miRNA gene | Mir6374 | microRNA 6374 |
| 6 | 83417439 | 83420991 |  |  | MGI:1925345 | unclassified gene | 5430434F05Rik | RIKEN cDNA 5430434F05 gene |
| 6 | 83441755 | 83448322 | 35.94 | + | MGI:2444713 | unclassified non-coding RNA gene | B230319C09Rik | RIKEN cDNA B230319C09 gene |
| 6 | 83480217 | 83506969 | 35.94 | - | MGI:1351602 | protein coding gene | Dguok | deoxyguanosine kinase |
| 6 | 83508654 | 83508745 |  | - | MGI:5455515 | miRNA gene | Gm25738 | predicted gene, 25738 |
| 6 | 83512905 | 83536265 | 35.94 | - | MGI:104589 | protein coding gene | Actg2 | actin, gamma 2, smooth muscle, enteric |
| 6 | 83543206 | 83572504 | 35.94 | - | MGI:1917777 | protein coding gene | Stambp | STAM binding protein |
| 6 | 83568039 | 83588121 |  | + | MGI:5434639 | unclassified non-coding RNA gene | Gm21284 | predicted gene, 21284 |
| 6 | 83591821 | 83592143 |  | - | MGI:5454767 | unclassified non-coding RNA gene | Gm24990 | predicted gene, 24990 |
| 6 | 83644542 | 83656187 | 35.94 | - | MGI:1859834 | protein coding gene | Clec4f | C-type lectin domain family 4, member f |
| 6 | 83671207 | 83677857 | 35.94 | - | MGI:2180021 | protein coding gene | Cd207 | CD207 antigen |
| 6 | 83692806 | 83712201 | 35.94 | - | MGI:3583301 | unclassified non-coding RNA gene | Vax2os | ventral anterior homeobox 2, opposite strand |
| 6 | 83702491 | 83702677 |  | + | MGI:5531154 | unclassified non-coding RNA gene | Gm27772 | predicted gene, 27772 |
| 6 | 83703518 | 83703983 |  | + | MGI:5531013 | unclassified non-coding RNA gene | Gm27631 | predicted gene, 27631 |
| 6 | 83710739 | 83710932 |  | + | MGI:5531275 | unclassified non-coding RNA gene | Gm27893 | predicted gene, 27893 |
| 6 | 83711264 | 83738313 | 35.94 | + | MGI:1346018 | protein coding gene | Vax2 | ventral anterior homeobox 2 |
| 6 | 83721296 | 83721392 |  | + | MGI:5454831 | snRNA gene | Gm25054 | predicted gene, 25054 |
| 6 | 83743017 | 83758809 | 35.94 | + | MGI:103285 | protein coding gene | Atp6v1b1 | ATPase, H+ transporting, lysosomal V1 subunit B1 |
| 6 | 83761756 | 83762865 | 35.94 | - | MGI:1915184 | unclassified non-coding RNA gene | 1700124L16Rik | RIKEN cDNA 1700124L16 gene |
| 6 | 83762646 | 83768326 | 35.94 | + | MGI:1922555 | protein coding gene | Ankrd53 | ankyrin repeat domain 53 |
| 6 | 83768885 | 83775813 | 35.94 | - | MGI:1096575 | protein coding gene | Tex261 | testis expressed gene 261 |
| 6 | 83794982 | 83803420 | 35.96 | + | MGI:1860418 | protein coding gene | Nagk | N-acetylglucosamine kinase |
| 6 | 83805401 | 83831741 | 35.97 | - | MGI:2386865 | protein coding gene | Paip2b | poly(A) binding protein interacting protein 2B |
| 6 | 83836937 | 83837066 |  | - | MGI:5452791 | snoRNA gene | Gm23014 | predicted gene, 23014 |
| 6 | 83914353 | 83986869 | 36.06 | + | MGI:1203484 | protein coding gene | Zfml | zinc finger, matrin-like |
| 6 | 84008590 | 84211060 | 36.14 | + | MGI:1349385 | protein coding gene | Dysf | dysferlin |
| 6 | 84101206 | 84107002 | 36.22 | - | MGI:3705288 | unclassified gene | Gm15475 | predicted gene 15475 |
| 6 | 84412942 | 84423838 |  | - | MGI:3642570 | unclassified non-coding RNA gene | Gm10445 | predicted gene 10445 |
| 6 | 84535875 | 84536320 |  |  | MGI:1925267 | unclassified gene | 4930504D19Rik | RIKEN cDNA 4930504D19 gene |
| 6 | 84571414 | 84593908 | 36.45 | - | MGI:2176159 | protein coding gene | Cyp26b1 | cytochrome P450, family 26, subfamily b, polypeptide 1 |
| 6 | 84618487 | 85069513 | 36.55 | - | MGI:1923164 | protein coding gene | Exoc6b | exocyst complex component 6B |
| 6 | 84898157 | 84898627 |  |  | MGI:3528153 | unclassified gene | AW125320 | cDNA sequence AW125320 |
| 6 | 85066183 | 85066437 |  | + | MGI:5456130 | unclassified non-coding RNA gene | Gm26353 | predicted gene, 26353 |
| 6 | 85111416 | 85126125 | 37.13 | - | MGI:3647625 | protein coding gene | Gm5878 | predicted gene 5878 |
| 6 | 85133678 | 85137766 | 37.15 | - | MGI:103078 | protein coding gene | Spr | sepiapterin reductase |
| 6 | 85150336 | 85150451 |  | + | MGI:5452670 | snoRNA gene | Gm22893 | predicted gene, 22893 |
| 6 | 85187438 | 85204462 | 37.21 | + | MGI:95387 | protein coding gene | Emx1 | empty spiracles homeobox 1 |
| 6 | 85213051 | 85333422 | 37.23 | - | MGI:2137681 | protein coding gene | Sfxn5 | sideroflexin 5 |
| 6 | 85243862 | 85246656 |  |  | MGI:2444182 | unclassified gene | D930014O13Rik | RIKEN cDNA D930014O13 gene |
| 6 | 85334963 | 85374634 | 37.35 | - | MGI:1098586 | protein coding gene | Rab11fip5 | RAB11 family interacting protein 5 (class I) |
| 6 | 85336292 | 85336373 | 37.35 | - | MGI:3629673 | miRNA gene | Mir705 | microRNA 705 |
| 6 | 85376201 | 85377906 |  |  | MGI:1918407 | unclassified gene | 4933423K11Rik | RIKEN cDNA 4933423K11 gene |
| 6 | 85423886 | 85428877 | 37.44 | + | MGI:3053002 | protein coding gene | Noto | notochord homolog (Xenopus laevis) |
| 6 | 85431989 | 85446435 | 37.44 | + | MGI:108048 | protein coding gene | Smyd5 | SET and MYND domain containing 5 |
| 6 | 85446810 | 85451970 | 37.46 | - | MGI:1920577 | protein coding gene | Pradc1 | protease-associated domain containing 1 |
| 6 | 85451505 | 85468477 | 37.46 | + | MGI:107184 | protein coding gene | Cct7 | chaperonin containing Tcp1, subunit 7 (eta) |
| 6 | 85469574 | 85502994 | 37.48 | - | MGI:1261912 | protein coding gene | Fbxo41 | F-box protein 41 |
| 6 | 85511122 | 85513542 | 37.48 | - | MGI:99252 | protein coding gene | Egr4 | early growth response 4 |
| 6 | 85587531 | 85702753 | 37.48 | + | MGI:1934606 | protein coding gene | Alms1 | Alstrom syndrome 1 |
| 6 | 85706727 | 85713205 | 37.48 | - | MGI:3782661 | protein coding gene | Gm4477 | predicted gene 4477 |
| 6 | 85743515 | 85743609 |  | - | MGI:5530699 | miRNA gene | Gm27317 | predicted gene, 27317 |
| 6 | 85760649 | 85765744 | 37.48 | - | MGI:2136449 | protein coding gene | Cml3 | camello-like 3 |
| 6 | 85790140 | 85790234 |  | - | MGI:5562769 | miRNA gene | Mir6375 | microRNA 6375 |
| 6 | 85808024 | 85820954 | 37.48 | - | MGI:3779382 | protein coding gene | Gm11128 | predicted gene 11128 |
| 6 | 85817221 | 85820972 | 37.48 | - | MGI:1916299 | protein coding gene | Cml5 | camello-like 5 |
| 6 | 85830388 | 85832082 | 37.49 | - | MGI:1915646 | protein coding gene | Nat8 | N-acetyltransferase 8 (GCN5-related, putative) |
| 6 | 85865422 | 85869158 | 37.51 | - | MGI:2136446 | protein coding gene | Cml2 | camello-like 2 |
| 6 | 85899051 | 85904884 | 37.52 | - | MGI:1922791 | protein coding gene | 1700019G17Rik | RIKEN cDNA 1700019G17 gene |
| 6 | 85910154 | 85915687 | 37.53 | - | MGI:1913366 | protein coding gene | Cml1 | camello-like 1 |
| 6 | 85915738 | 85930284 | 37.53 | + | MGI:1917036 | protein coding gene | Tprkb | Tp53rk binding protein |
| 6 | 85932681 | 85933379 | 37.54 | - | MGI:3644831 | protein coding gene | Nat8b | N-acetyltransferase 8B |
| 6 | 85942268 | 85961667 | 37.54 | - | MGI:1919352 | protein coding gene | Dusp11 | dual specificity phosphatase 11 (RNA/RNP complex 1-interacting) |
| 6 | 85961696 | 85966020 | 37.55 | + | MGI:5011450 | unclassified non-coding RNA gene | Gm19265 | predicted gene, 19265 |
| 6 | 86004384 | 86009345 |  |  | MGI:1922605 | unclassified non-coding RNA gene | 4930553P18Rik | RIKEN cDNA 4930553P18 gene |
| 6 | 86017191 | 86020996 | 37.55 | + | MGI:1349421 | protein coding gene | Figla | folliculogenesis specific basic helix-loop-helix |
| 6 | 86078084 | 86119555 | 37.55 | + | MGI:87919 | protein coding gene | Add2 | adducin 2 (beta) |
| 6 | 86195251 | 86275449 | 37.62 | + | MGI:98724 | protein coding gene | Tgfa | transforming growth factor alpha |
| 6 | 86365683 | 86370057 | 37.69 | + | MGI:1913738 | protein coding gene | Fam136a | family with sequence similarity 136, member A |
| 6 | 86371583 | 86378902 | 37.69 | + | MGI:1915261 | protein coding gene | Snrpg | small nuclear ribonucleoprotein polypeptide G |
| 6 | 86372538 | 86375285 | 37.69 | - | MGI:5011458 | unclassified non-coding RNA gene | Gm19273 | predicted gene, 19273 |
| 6 | 86386006 | 86397150 | 37.69 | - | MGI:1914131 | protein coding gene | Pcyox1 | prenylcysteine oxidase 1 |
| 6 | 86403893 | 86403963 |  | + | MGI:4413869 | tRNA gene | n-Tg8 | nuclear encoded tRNA glycine 8 (anticodon CCC) |
| 6 | 86404219 | 86433403 | 37.69 | + | MGI:107914 | protein coding gene | Tia1 | cytotoxic granule-associated RNA binding protein 1 |
| 6 | 86438374 | 86473500 | 37.69 | + | MGI:2141787 | protein coding gene | C87436 | expressed sequence C87436 |
| 6 | 86470805 | 86480830 | 37.7 | - | MGI:2444132 | unclassified gene | A430078I02Rik | RIKEN cDNA A430078I02 gene |
| 6 | 86483376 | 86488227 | 37.71 | - | MGI:1916897 | unclassified non-coding RNA gene | 2310040G24Rik | RIKEN cDNA 2310040G24 gene |
| 6 | 86524492 | 86526321 | 37.72 | - | MGI:1345635 | protein coding gene | Pcbp1 | poly(rC) binding protein 1 |
| 6 | 86527330 | 86564449 | 37.72 | + | MGI:1919262 | lincRNA gene | 1600020E01Rik | RIKEN cDNA 1600020E01 gene |
| 6 | 86574100 | 86574161 |  | - | MGI:5451961 | miRNA gene | Gm22184 | predicted gene, 22184 |
| 6 | 86628174 | 86629702 | 37.75 | + | MGI:1915105 | protein coding gene | Asprv1 | aspartic peptidase, retroviral-like 1 |
| 6 | 86647044 | 86669159 | 37.75 | - | MGI:96908 | protein coding gene | Mxd1 | MAX dimerization protein 1 |
| 6 | 86675221 | 86684522 | 37.75 | - | MGI:1913868 | protein coding gene | Snrnp27 | small nuclear ribonucleoprotein 27 (U4/U6.U5) |
| 6 | 86691768 | 86733378 | 37.75 | - | MGI:1345156 | protein coding gene | Gmcl1 | germ cell-less homolog 1 (Drosophila) |
| 6 | 86736840 | 86765910 | 37.75 | - | MGI:88030 | protein coding gene | Anxa4 | annexin A4 |
| 6 | 86848399 | 86849440 | 37.75 | - | MGI:1914420 | unclassified non-coding RNA gene | 2610306M01Rik | RIKEN cDNA 2610306M01 gene |
| 6 | 86849517 | 86991864 | 37.75 | + | MGI:1098687 | protein coding gene | Aak1 | AP2 associated kinase 1 |
| 6 | 87009236 | 87028461 | 37.76 | + | MGI:1913290 | protein coding gene | Nfu1 | NFU1 iron-sulfur cluster scaffold homolog (S. cerevisiae) |
| 6 | 87042846 | 87092197 | 37.81 | + | MGI:95698 | protein coding gene | Gfpt1 | glutamine fructose-6-phosphate transaminase 1 |
| 6 | 87104826 | 87112251 | 37.9 | + | MGI:1261919 | protein coding gene | D6Ertd527e | DNA segment, Chr 6, ERATO Doi 527, expressed |
| 6 | 87133854 | 87335775 | 37.94 | - | MGI:1916788 | protein coding gene | Antxr1 | anthrax toxin receptor 1 |
| 6 | 87281149 | 87281260 | 37.96 | + | MGI:4422028 | rRNA gene | n-R5s164 | nuclear encoded rRNA 5S 164 |
| 6 | 87321557 | 87322338 |  |  | MGI:1924658 | unclassified gene | 9530013L04Rik | RIKEN cDNA 9530013L04 gene |
| 6 | 87345653 | 87350915 | 37.96 | - | MGI:1913533 | protein coding gene | Gkn1 | gastrokine 1 |
| 6 | 87373365 | 87379494 | 37.96 | + | MGI:1913534 | protein coding gene | Gkn2 | gastrokine 2 |
| 6 | 87383268 | 87388935 | 37.96 | - | MGI:1916138 | protein coding gene | Gkn3 | gastrokine 3 |
| 6 | 87429002 | 87434512 | 37.96 | + | MGI:1338820 | protein coding gene | Bmp10 | bone morphogenetic protein 10 |
| 6 | 87458545 | 87533259 | 38.01 | - | MGI:2443687 | protein coding gene | Arhgap25 | Rho GTPase activating protein 25 |
| 6 | 87578593 | 87590701 | 38.28 | - | MGI:1929676 | protein coding gene | Prokr1 | prokineticin receptor 1 |
| 6 | 87628429 | 87672168 | 38.52 | - | MGI:1919353 | protein coding gene | Aplf | aprataxin and PNKP like factor |
| 6 | 87672382 | 87681024 | 38.74 | + | MGI:2443140 | unclassified non-coding RNA gene | E230015B07Rik | RIKEN cDNA E230015B07 gene |
| 6 | 87675593 | 87690847 | 38.75 | - | MGI:3586838 | lincRNA gene | 1810020O05Rik | Riken cDNA 1810020O05 gene |
| 6 | 87707198 | 87722084 | 38.91 | - | MGI:5011803 | protein coding gene | Gm19618 | predicted gene, 19618 |
| 6 | 87727241 | 87727333 |  | + | MGI:5453178 | miRNA gene | Gm23401 | predicted gene, 23401 |
| 6 | 87730869 | 87755908 | 39.02 | + | MGI:3611451 | protein coding gene | Efcc1 | EF hand and coiled-coil domain containing 1 |
| 6 | 87778136 | 87779762 | 39.13 | + | MGI:1860137 | protein coding gene | Gp9 | glycoprotein 9 (platelet) |
| 6 | 87788853 | 87812164 | 39.13 | - | MGI:1917084 | protein coding gene | Rab43 | RAB43, member RAS oncogene family |
| 6 | 87805471 | 87806335 |  |  | MGI:1924996 | unclassified gene | 9930120I10Rik | RIKEN cDNA 9930120I10 gene |
| 6 | 87818447 | 87838759 | 39.13 | - | MGI:1923310 | protein coding gene | Isy1 | ISY1 splicing factor homolog (S. cerevisiae) |
| 6 | 87830038 | 87832865 |  |  | MGI:2442518 | unclassified gene | B130021K23Rik | RIKEN cDNA B130021K23 gene |
| 6 | 87843082 | 87851106 | 39.13 | - | MGI:88431 | protein coding gene | Cnbp | cellular nucleic acid binding protein |
| 6 | 87887814 | 87913595 | 39.13 | + | MGI:1858696 | protein coding gene | Copg1 | coatomer protein complex, subunit gamma 1 |
| 6 | 87908843 | 87914459 |  | - | MGI:5477130 | lincRNA gene | Gm26636 | predicted gene, 26636 |
| 6 | 87913935 | 87936629 | 39.13 | + | MGI:1914053 | protein coding gene | Hmces | 5-hydroxymethylcytosine (hmC) binding, ES cell specific |
| 6 | 87980421 | 87981637 | 39.13 | - | MGI:2685307 | protein coding gene | H1fx | H1 histone family, member X |
| 6 | 87980883 | 88005471 | 39.13 | + | MGI:3648213 | protein coding gene | Gm5577 | predicted gene 5577 |
| 6 | 87993135 | 87994178 |  |  | MGI:1921328 | unclassified gene | 4933412L11Rik | RIKEN cDNA 4933412L11 gene |
| 6 | 87999106 | 88045270 | 39.13 | - | MGI:105068 | protein coding gene | Rab7 | RAB7, member RAS oncogene family |
| 6 | 88084473 | 88105304 | 39.13 | + | MGI:98084 | protein coding gene | Rpn1 | ribophorin I |
| 6 | 88104128 | 88104258 |  | - | MGI:5531160 | miRNA gene | Mir6376 | microRNA 6376 |
| 6 | 88198664 | 88207032 | 39.2 | + | MGI:95662 | protein coding gene | Gata2 | GATA binding protein 2 |
| 6 | 88222268 | 88223257 | 39.25 | + | MGI:1922801 | protein coding gene | Dnajb8 | DnaJ (Hsp40) homolog, subfamily B, member 8 |
| 6 | 88222602 | 88225678 | 39.25 | - | MGI:1920567 | unclassified gene | 1700031F10Rik | RIKEN cDNA 1700031F10 gene |
| 6 | 88257334 | 88446539 | 39.32 | - | MGI:2137092 | protein coding gene | Eefsec | eukaryotic elongation factor, selenocysteine-tRNA-specific |
| 6 | 88405977 | 88406301 |  | + | MGI:5454955 | unclassified non-coding RNA gene | Gm25178 | predicted gene, 25178 |
| 6 | 88465409 | 88497572 | 39.51 | + | MGI:1928760 | protein coding gene | Ruvbl1 | RuvB-like protein 1 |
| 6 | 88503601 | 88518905 | 39.51 | - | MGI:1858417 | protein coding gene | Sec61a1 | Sec61 alpha 1 subunit (S. cerevisiae) |
| 6 | 88547340 | 88637950 | 39.51 | - | MGI:1918481 | protein coding gene | Kbtbd12 | kelch repeat and BTB (POZ) domain containing 12 |
| 6 | 88716424 | 88724501 |  | - | MGI:5477082 | lincRNA gene | Gm26588 | predicted gene, 26588 |
| 6 | 88724412 | 88828360 | 39.51 | + | MGI:1346042 | protein coding gene | Mgll | monoglyceride lipase |
| 6 | 88835915 | 88841935 | 39.6 | - | MGI:1933148 | protein coding gene | Abtb1 | ankyrin repeat and BTB (POZ) domain containing 1 |
| 6 | 88842558 | 88875044 | 39.6 | - | MGI:2442488 | protein coding gene | Podxl2 | podocalyxin-like 2 |
| 6 | 88842854 | 88847274 | 39.61 | + | MGI:3641714 | unclassified non-coding RNA gene | Gm15612 | predicted gene 15612 |
| 6 | 88883475 | 88898780 | 39.64 | - | MGI:105380 | protein coding gene | Mcm2 | minichromosome maintenance deficient 2 mitotin (S. cerevisiae) |
| 6 | 88902251 | 88912238 | 39.65 | + | MGI:1345190 | protein coding gene | Tpra1 | transmembrane protein, adipocyte asscociated 1 |
| 6 | 89096110 | 89110030 | 39.79 | + | MGI:3026922 | protein coding gene | 4933427D06Rik | RIKEN cDNA 4933427D06 gene |
| 6 | 89141005 | 89147385 | 39.81 | + | MGI:3584270 | protein coding gene | Gm1965 | predicted gene 1965 |
| 6 | 89184672 | 89186025 | 39.84 | - | MGI:3645279 | lincRNA gene | Gm6507 | predicted gene 6507 |
| 6 | 89211165 | 89216237 | 39.85 | - | MGI:2685685 | protein coding gene | Gm839 | predicted gene 839 |
| 6 | 89249468 | 89249602 |  | - | MGI:5455738 | rRNA gene | Gm25961 | predicted gene, 25961 |
| 6 | 89269510 | 89285724 |  |  | MGI:1922381 | unclassified gene | 4930512J16Rik | RIKEN cDNA 4930512J16 gene |
| 6 | 89304630 | 89327621 |  | + | MGI:5477305 | lincRNA gene | Gm26811 | predicted gene, 26811 |
| 6 | 89316316 | 89362613 | 39.91 | - | MGI:107685 | protein coding gene | Plxna1 | plexin A1 |
| 6 | 89383146 | 89595652 | 39.91 | - | MGI:1913348 | protein coding gene | Chchd6 | coiled-coil-helix-coiled-coil-helix domain containing 6 |
| 6 | 89643988 | 89675529 | 40.01 | + | MGI:2386711 | protein coding gene | Txnrd3 | thioredoxin reductase 3 |
| 6 | 89714203 | 89715135 | 40.02 | + | MGI:2148518 | protein coding gene | Vmn1r40 | vomeronasal 1 receptor 40 |
| 6 | 89746479 | 89747414 | 40.02 | + | MGI:2148520 | protein coding gene | Vmn1r41 | vomeronasal 1 receptor 41 |
| 6 | 89844518 | 89845615 | 35.94 | + | MGI:2148511 | protein coding gene | Vmn1r42 | vomeronasal 1 receptor 42 |
| 6 | 89869513 | 89870502 | 37.96 | - | MGI:2148510 | protein coding gene | Vmn1r43 | vomeronasal 1 receptor 43 |
| 6 | 89893177 | 89894284 | 37.48 | - | MGI:2148517 | protein coding gene | Vmn1r44 | vomeronasal 1 receptor 44 |
| 6 | 89931649 | 89940507 | 39.13 | - | MGI:1333762 | protein coding gene | Vmn1r45 | vomeronasal 1 receptor 45 |
| 6 | 89976171 | 89977100 | 35.94 | - | MGI:2148519 | protein coding gene | Vmn1r46 | vomeronasal 1 receptor 46 |
| 6 | 90021888 | 90022820 | 39.51 | - | MGI:2148509 | protein coding gene | Vmn1r47 | vomeronasal 1 receptor 47 |
| 6 | 90035933 | 90036841 | 40.16 | + | MGI:2148508 | protein coding gene | Vmn1r48 | vomeronasal 1 receptor 48 |
| 6 | 90072086 | 90073018 | 37.46 | - | MGI:1344384 | protein coding gene | Vmn1r49 | vomeronasal 1, receptor 49 |
| 6 | 90107275 | 90108207 | 40.54 | + | MGI:2148515 | protein coding gene | Vmn1r50 | vomeronasal 1 receptor 50 |
| 6 | 90122643 | 90130990 | 35.94 | + | MGI:1333759 | protein coding gene | Vmn1r51 | vomeronasal 1 receptor 51 |
| 6 | 90178716 | 90179645 | 39.25 | + | MGI:2148512 | protein coding gene | Vmn1r52 | vomeronasal 1 receptor 52 |
| 6 | 90202793 | 90203682 | 35.94 | - | MGI:2148513 | protein coding gene | V1ra8 | vomeronasal 1 receptor, A8 |
| 6 | 90223317 | 90224438 | 36.55 | - | MGI:2148516 | protein coding gene | Vmn1r53 | vomeronasal 1 receptor 53 |
| 6 | 90269106 | 90270053 | 41.05 | - | MGI:2148514 | protein coding gene | Vmn1r54 | vomeronasal 1 receptor 54 |
| 6 | 90301219 | 90305449 | 44.69 | - | MGI:2679261 | protein coding gene | BC048671 | cDNA sequence BC048671 |
| 6 | 90308351 | 90325185 | 35.94 | + | MGI:1919047 | protein coding gene | Chst13 | carbohydrate (chondroitin 4) sulfotransferase 13 |
| 6 | 90333289 | 90364551 | 40.16 | + | MGI:2385332 | protein coding gene | Uroc1 | urocanase domain containing 1 |
| 6 | 90369492 | 90403490 | 39.6 | - | MGI:1933108 | protein coding gene | Zxdc | ZXD family zinc finger C |
| 6 | 90386806 | 90389356 | 46.95 | - | MGI:1924668 | unclassified gene | C030015A19Rik | RIKEN cDNA C030015A19 gene |
| 6 | 90403736 | 90428797 | 37.48 | - | MGI:2141635 | protein coding gene | Ccdc37 | coiled-coil domain containing 37 |
| 6 | 90462576 | 90475238 | 40.16 | - | MGI:1929988 | protein coding gene | Klf15 | Kruppel-like factor 15 |
| 6 | 90486427 | 90600203 | 44.88 | - | MGI:1340024 | protein coding gene | Aldh1l1 | aldehyde dehydrogenase 1 family, member L1 |
| 6 | 90596685 | 90599948 | 37.69 | - | MGI:3783199 | unclassified non-coding RNA gene | Gm15756 | predicted gene 15756 |
| 6 | 90604725 | 90646412 | 45.98 | - | MGI:1918949 | protein coding gene | Slc41a3 | solute carrier family 41, member 3 |
| 6 | 90662346 | 90764141 | 37.75 | + | MGI:1196356 | protein coding gene | Iqsec1 | IQ motif and Sec7 domain 1 |
| 6 | 90681370 | 90681520 | 41.41 | - | MGI:5454593 | miRNA gene | Gm24816 | predicted gene, 24816 |
| 6 | 90777741 | 90782080 | 37.94 | - | MGI:2444127 | unclassified gene | E130314K07Rik | RIKEN cDNA E130314K07 gene |
| 6 | 90857537 | 90857632 | 37.53 | + | MGI:5454962 | snoRNA gene | Gm25185 | predicted gene, 25185 |
| 6 | 91013068 | 91116829 | 35.94 | + | MGI:1859555 | protein coding gene | Nup210 | nucleoporin 210 |
| 6 | 91156665 | 91174692 | 35.94 | - | MGI:2385252 | protein coding gene | Hdac11 | histone deacetylase 11 |
| 6 | 91158713 | 91159256 | 40.13 | - | MGI:3705150 | unclassified gene | Gm14573 | predicted gene 14573 |
| 6 | 91206783 | 91210043 | 35.94 | + | MGI:1926044 | unclassified gene | 4930402H05Rik | RIKEN cDNA 4930402H05 gene |
| 6 | 91212455 | 91272540 | 37.54 | - | MGI:95488 | protein coding gene | Fbln2 | fibulin 2 |
| 6 | 91363981 | 91411363 | 38.52 | - | MGI:98961 | protein coding gene | Wnt7a | wingless-type MMTV integration site family, member 7A |
| 6 | 91430460 | 91440854 | 40.53 | - | MGI:1922150 | lincRNA gene | 4930471M09Rik | RIKEN cDNA 4930471M09 gene |
| 6 | 91440987 | 91441755 | 46.82 | - | MGI:1917048 | unclassified non-coding RNA gene | 1810044D09Rik | RIKEN cDNA 1810044D09 gene |
| 6 | 91464276 | 91473423 | 41.54 | + | MGI:1919420 | protein coding gene | Chchd4 | coiled-coil-helix-coiled-coil-helix domain containing 4 |
| 6 | 91473703 | 91488459 | 37.15 | - | MGI:1921372 | protein coding gene | Tmem43 | transmembrane protein 43 |
| 6 | 91489305 | 91515884 | 35.94 | + | MGI:103557 | protein coding gene | Xpc | xeroderma pigmentosum, complementation group C |
| 6 | 91515928 | 91522625 | 45.05 | + | MGI:1914928 | protein coding gene | Lsm3 | LSM3 homolog, U6 small nuclear RNA associated (S. cerevisiae) |
| 6 | 91599235 | 91605514 | 35.94 | - | MGI:5011647 | unclassified non-coding RNA gene | Gm19462 | predicted gene, 19462 |
| 6 | 91684067 | 91759063 | 35.94 | - | MGI:98488 | protein coding gene | Slc6a6 | solute carrier family 6 (neurotransmitter transporter, taurine), member 6 |
| 6 | 91689932 | 91691607 | 39.13 | - | MGI:1921977 | unclassified gene | 4930517G19Rik | RIKEN cDNA 4930517G19 gene |
| 6 | 91761509 | 91827250 | 39.64 | - | MGI:2681173 | protein coding gene | Grip2 | glutamate receptor interacting protein 2 |
| 6 | 91878053 | 91899843 | 35.94 | + | MGI:2444652 | protein coding gene | Ccdc174 | coiled-coil domain containing 174 |
| 6 | 91914759 | 91950640 | 35.94 | + | MGI:2685917 | protein coding gene | 4930590J08Rik | RIKEN cDNA 4930590J08 gene |
| 6 | 91978878 | 92076004 | 35.94 | + | MGI:2443369 | protein coding gene | Fgd5 | FYVE, RhoGEF and PH domain containing 5 |
| 6 | 92012394 | 92016117 | 43.15 | - | MGI:1923066 | unclassified gene | 4930466I24Rik | RIKEN cDNA 4930466I24 gene |
| 6 | 92091390 | 92173057 | 37.44 | + | MGI:1352466 | protein coding gene | Nr2c2 | nuclear receptor subfamily 2, group C, member 2 |
| 6 | 92165991 | 92167015 | 35.94 | - | MGI:1924500 | unclassified gene | 9430019H13Rik | RIKEN cDNA 9430019H13 gene |
| 6 | 92169525 | 92184033 | 35.94 | - | MGI:1928140 | protein coding gene | Mrps25 | mitochondrial ribosomal protein S25 |
| 6 | 92186712 | 92214925 | 37.35 | - | MGI:1925537 | protein coding gene | Zfyve20 | zinc finger, FYVE domain containing 20 |
| 6 | 92242061 | 92244650 | 37.75 | + | MGI:98823 | protein coding gene | Trh | thyrotropin releasing hormone |
| 6 | 92271414 | 92271572 | 40.16 | - | MGI:5011660 | protein coding gene | Gm19475 | predicted gene, 19475 |
| 6 | 92370908 | 92567366 | 36.06 | + | MGI:1925144 | protein coding gene | Prickle2 | prickle homolog 2 (Drosophila) |
| 6 | 92772699 | 92943492 | 37.48 | - | MGI:1916320 | protein coding gene | Adamts9 | a disintegrin-like and metallopeptidase (reprolysin type) with thrombospondin type 1 motif, 9 |
| 6 | 92816478 | 92847174 | 37.9 | + | MGI:1921766 | protein coding gene | A730049H05Rik | RIKEN cDNA A730049H05 gene |
| 6 | 92869357 | 92884411 | 35.94 | - | MGI:3783179 | protein coding gene | Gm15737 | predicted gene 15737 |
| 6 | 92940582 | 93111749 | 35.94 | + | MGI:1924659 | lincRNA gene | 9530026P05Rik | RIKEN cDNA 9530026P05 gene |
| 6 | 92963853 | 92964660 | 40.55 | - | MGI:2441979 | unclassified gene | D630004L18Rik | RIKEN cDNA D630004L18 gene |
| 6 | 93335458 | 93335588 | 37.46 | + | MGI:5454871 | snoRNA gene | Gm25094 | predicted gene, 25094 |
| 6 | 93509573 | 93509702 | 39.91 | - | MGI:5452089 | snoRNA gene | Gm22312 | predicted gene, 22312 |
| 6 | 93675455 | 94283322 | 37.69 | + | MGI:1203522 | protein coding gene | Magi1 | membrane associated guanylate kinase, WW and PDZ domain containing 1 |
| 6 | 93793772 | 93793877 | 35.94 | + | MGI:5452812 | snRNA gene | Gm23035 | predicted gene, 23035 |
| 6 | 93890615 | 93890750 | 46.22 | + | MGI:5452617 | snoRNA gene | Gm22840 | predicted gene, 22840 |
| 6 | 93917764 | 93920776 | 42.7 | - | MGI:3028056 | unclassified gene | B430316J06Rik | RIKEN cDNA B430316J06 gene |
| 6 | 93988801 | 93989738 | 44.63 | - | MGI:1924419 | unclassified gene | 8030459D09Rik | RIKEN cDNA 8030459D09 gene |
| 6 | 94132922 | 94237443 | 40.02 | + | MGI:1925428 | unclassified gene | 4930511A08Rik | RIKEN cDNA 4930511A08 gene |
| 6 | 94500314 | 94604648 | 40.02 | - | MGI:1914832 | protein coding gene | Slc25a26 | solute carrier family 25 (mitochondrial carrier, phosphate carrier), member 26 |
| 6 | 94604529 | 94700158 | 46.93 | + | MGI:107935 | protein coding gene | Lrig1 | leucine-rich repeats and immunoglobulin-like domains 1 |
| 6 | 94606364 | 94606415 | 35.94 | - | MGI:5531254 | miRNA gene | Mir7041 | microRNA 7041 |
| 6 | 94823733 | 94823805 | 35.94 | + | MGI:5453141 | miRNA gene | Gm23364 | predicted gene, 23364 |
| 6 | 94826781 | 94826923 | 37.44 | + | MGI:5454378 |  | Gm24601 | predicted gene, 24601 |
| 6 | 94943834 | 94951545 | 40.8 | - | MGI:1926023 | unclassified non-coding RNA gene | 4930511E03Rik | RIKEN cDNA 4930511E03 gene |
| 6 | 95092012 | 95095080 | 37.69 | + | MGI:1920900 | unclassified gene | 2410024F20Rik | RIKEN cDNA 2410024F20 gene |
| 6 | 95100201 | 95100307 | 44.93 | - | MGI:5456278 | snRNA gene | Gm26501 | predicted gene, 26501 |
| 6 | 95114017 | 95117631 | 40.02 | - | MGI:1919467 | unclassified gene | Kbtbd8os | kelch repeat and BTB (POZ) domain containing 8, opposite strand |
| 6 | 95117240 | 95129718 | 40.02 | + | MGI:2661430 | protein coding gene | Kbtbd8 | kelch repeat and BTB (POZ) domain containing 8 |
| 6 | 95255726 | 95333993 | 40.1 | + | MGI:3041245 | unclassified non-coding RNA gene | AY512915 | cDNA sequence AY512915 |
| 6 | 95322112 | 95322210 | 40.02 | + | MGI:3642207 | protein coding gene | Gm10234 | predicted gene 10234 |
| 6 | 95474134 | 95718837 | 40.02 | + | MGI:1306824 | protein coding gene | Suclg2 | succinate-Coenzyme A ligase, GDP-forming, beta subunit |
| 6 | 96035431 | 96036590 | 36.45 | - | MGI:1925169 | unclassified gene | A330102K18Rik | RIKEN cDNA A330102K18 gene |
| 6 | 96113154 | 96657198 | 39.13 | - | MGI:2443695 | protein coding gene | Fam19a1 | family with sequence similarity 19, member A1 |
| 6 | 96116422 | 96117263 | 41.03 | - | MGI:1920261 | unclassified gene | 2900060L22Rik | RIKEN cDNA 2900060L22 gene |
| 6 | 96164497 | 96166243 | 40.16 | + | MGI:1925732 | protein coding gene | 1700123L14Rik | RIKEN cDNA 1700123L14 gene |
| 6 | 96469435 | 96469531 | 35.94 | + | MGI:5452748 | snRNA gene | Gm22971 | predicted gene, 22971 |
| 6 | 96560761 | 96560864 | 37.51 | - | MGI:5455788 | snRNA gene | Gm26011 | predicted gene, 26011 |
| 6 | 96831209 | 97060411 | 39.32 | - | MGI:2444563 | protein coding gene | Fam19a4 | family with sequence similarity 19, member A4 |
| 6 | 97110024 | 97149182 | 37.23 | - | MGI:2141669 | protein coding gene | Eogt | EGF domain-specific O-linked N-acetylglucosamine (GlcNAc) transferase |
| 6 | 97155266 | 97179124 | 40.02 | - | MGI:2684999 | protein coding gene | Tmf1 | TATA element modulatory factor 1 |
| 6 | 97162817 | 97162947 | 40.02 | - | MGI:5455952 | snoRNA gene | Gm26175 | predicted gene, 26175 |
| 6 | 97183816 | 97205642 | 40.04 | + | MGI:1341217 | protein coding gene | Uba3 | ubiquitin-like modifier activating enzyme 3 |
| 6 | 97210689 | 97233315 | 40.05 | + | MGI:1929501 | protein coding gene | Arl6ip5 | ADP-ribosylation factor-like 6 interacting protein 5 |
| 6 | 97238528 | 97252780 | 40.02 | + | MGI:2444169 | protein coding gene | Lmod3 | leiomodin 3 (fetal) |
| 6 | 97286867 | 97617541 | 40.07 | - | MGI:2141794 | protein coding gene | Frmd4b | FERM domain containing 4B |
| 6 | 97547438 | 97547567 | 40.02 | + | MGI:5455629 | snoRNA gene | Gm25852 | predicted gene, 25852 |
| 6 | 97572076 | 97572238 | 40.02 | + | MGI:5453287 | snRNA gene | Gm23510 | predicted gene, 23510 |
| 6 | 97619678 | 97620415 | 35.81 | + | MGI:1925782 | unclassified gene | C030006F08Rik | RIKEN cDNA C030006F08 gene |
| 6 | 97730439 | 97730545 | 37.48 | + | MGI:5454342 | snRNA gene | Gm24565 | predicted gene, 24565 |
| 6 | 97790512 | 97790654 | 40.01 | + | MGI:5453480 | snoRNA gene | Gm23703 | predicted gene, 23703 |
| 6 | 97807058 | 98021349 | 39.6 | - | MGI:104554 | protein coding gene | Mitf | microphthalmia-associated transcription factor |
| 6 | 98238014 | 98342754 | 35.94 | + | MGI:2685611 | protein coding gene | Gm765 | predicted gene 765 |
| 6 | 98535246 | 98552109 | 35.94 | + | MGI:1925309 | unclassified gene | 4930595L18Rik | RIKEN cDNA 4930595L18 gene |
| 6 | 98558739 | 98558831 | 41.0 | + | MGI:5454164 | miRNA gene | Gm24387 | predicted gene, 24387 |
| 6 | 98693611 | 98697673 | 38.01 | - | MGI:1925058 | unclassified gene | A930015G24Rik | RIKEN cDNA A930015G24 gene |
| 6 | 98925338 | 99522721 | 44.67 | + | MGI:1914004 | protein coding gene | Foxp1 | forkhead box P1 |
| 6 | 98967701 | 98971926 | 44.92 | - | MGI:1921805 | unclassified gene | 9130401L11Rik | RIKEN cDNA 9130401L11 gene |
| 6 | 98997642 | 98998605 | 44.8 | - | MGI:1924976 | unclassified gene | 6030492E11Rik | RIKEN cDNA 6030492E11 gene |
| 6 | 99099255 | 99102944 | 40.9 | + | MGI:5011850 | unclassified gene | Gm19665 | predicted gene, 19665 |
| 6 | 99257484 | 99666797 | 44.32 | + | MGI:5313143 | protein coding gene | Gm20696 | predicted gene 20696 |
| 6 | 99384607 | 99388499 | 35.81 | + | MGI:5313152 | unclassified gene | Gm20705 | predicted gene 20705 |
| 6 | 99484591 | 99484702 | 35.97 | - | MGI:5452105 | miRNA gene | Gm22328 | predicted gene, 22328 |
| 6 | 99575891 | 99576137 | 40.15 | + | MGI:5454713 | unclassified non-coding RNA gene | Gm24936 | predicted gene, 24936 |
| 6 | 99625135 | 99666771 | 40.38 | + | MGI:1914142 | protein coding gene | Eif4e3 | eukaryotic translation initiation factor 4E member 3 |
| 6 | 99692679 | 99693818 | 40.12 | + | MGI:1202299 | protein coding gene | Gpr27 | G protein-coupled receptor 27 |
| 6 | 99711299 | 99726392 | 46.9 | + | MGI:1354178 | protein coding gene | Prok2 | prokineticin 2 |
| 6 | 99725771 | 99732553 | 39.79 | + | MGI:5477242 | lincRNA gene | Gm26748 | predicted gene, 26748 |
| 6 | 99893226 | 99893547 | 44.89 | - | MGI:5454025 | unclassified non-coding RNA gene | Gm24248 | predicted gene, 24248 |
| 6 | 100057020 | 114282207 | 39.13 | - | MGI:3761013 | heritable phenotypic marker | ssl | small swaying lethal |
| 6 | 100057020 | 114282207 | 45.67 | - | MGI:4437431 | heritable phenotypic marker | wblo | wobbly locomotion |
| 6 | 100143422 | 100143813 | 39.85 | - | MGI:1917515 | unclassified gene | 2010109P13Rik | RIKEN cDNA 2010109P13 gene |
| 6 | 100228565 | 100287358 | 40.94 | + | MGI:1929059 | protein coding gene | Rybp | RING1 and YY1 binding protein |
| 6 | 100494681 | 100494814 | 35.94 | - | MGI:5453011 | snoRNA gene | Gm23234 | predicted gene, 23234 |
| 6 | 100527400 | 100533426 | 37.54 | - | MGI:1920645 | unclassified non-coding RNA gene | 1700049E22Rik | RIKEN cDNA 1700049E22 gene |
| 6 | 100568257 | 100569731 | 44.42 | + | MGI:1917782 | unclassified gene | 5730433K22Rik | RIKEN cDNA 5730433K22 gene |
| 6 | 100573081 | 100671157 | 37.13 | - | MGI:1919421 | protein coding gene | Shq1 | SHQ1 homolog (S. cerevisiae) |
| 6 | 100704734 | 100805081 | 39.13 | + | MGI:2682940 | protein coding gene | Gxylt2 | glucoside xylosyltransferase 2 |
| 6 | 100791829 | 100793912 | 39.81 | + | MGI:5011877 | unclassified gene | Gm19692 | predicted gene, 19692 |
| 6 | 100833638 | 100868717 | 39.02 | + | MGI:3027896 | protein coding gene | Ppp4r2 | protein phosphatase 4, regulatory subunit 2 |
| 6 | 100922549 | 100923155 | 37.48 | - | MGI:1922624 | unclassified gene | 4930595O18Rik | RIKEN cDNA 4930595O18 gene |
| 6 | 101149609 | 101377897 | 37.48 | - | MGI:1933157 | protein coding gene | Pdzrn3 | PDZ domain containing RING finger 3 |
| 6 | 101198569 | 101210269 | 41.69 | + | MGI:5477405 | lincRNA gene | Gm26911 | predicted gene, 26911 |
| 6 | 101285825 | 101316547 | 41.03 | - | MGI:1925991 | unclassified gene | 9530086O07Rik | RIKEN cDNA 9530086O07 gene |
| 6 | 101796225 | 101801610 | 38.91 | - | MGI:3647145 | unclassified gene | Gm9871 | predicted gene 9871 |
