# Supplementary material for: Mapping genetic determinants of host susceptibility to Pseudomonas aeruginosa lung infection in mice
Source: BMC Genomics. 2016 May 11;17:351. doi: 10.1186/s12864-016-2676-4 (PMC4866434; doi:10.1186/s12864-016-2676-4)
Supplement: Additional file 5: — List of the most promising candidates genes for susceptibility to acute P. aeruginosa pulmonary infection. (DOCX 27 kb) [file 12864_2016_2676_MOESM5_ESM.docx]

**Table S4. List of the most promising candidates genes for susceptibility to acute *P. aeruginosa* pulmonary infection. A)** Candidate test genes are ranked based on the similarity with a set of known training genes. Each column contain the first sixteen most relevant genes obtained from their comparison to the training genes known to be correlated to Lung infection, Bacteraemia, Pneumonia, Sepsis, Chemotaxis, *P. aeruginosa* infection, Innate and Adaptive immune response, Pattern recognition receptor, Neutrophils, Complement, Cystic fibrosis, Modifier genes in Cystic fibrosis and chronic obstructive pulmonary disease (COPD). **B)** List of candidates obtained by filtering the list of genes in MGI database, based on different keywords (Lung, Infection, Respiratory AND infection) and gene ontology terms (Immune AND Response, Cytokines, Chemotaxis). The candidate genes highlighted by different colours are those taken in consideration for a putative role in host defence to infection by literature search.

**A**

| **Lung infection** | **Bacteraemia** | **Pneumonia** | **Sepsis** | ***Chemotaxis*** | ***P. aeruginosa infection*** | **Innate immune response** | **Adaptive immune**  **reponse** | **Pattern recognition receptor** | **Neutrophils** | **Complement** | **Cystic fibrosis** | **Modifier genes in CF** | **COPD** |
| --- | --- | --- | --- | --- | --- | --- | --- | --- | --- | --- | --- | --- | --- |
| **Cd207** | Lrig1 | Fbln2 | **Clec4f** | **Tacr1** | Anxa4 | **Gp9** | Lrig1 | Nr2c2 | **Tacr1** | Xpc | Fbln2 | Arl6ip5 | **Clec4f** |
| Bmp10 | Loxl3 | Bmp10 | Antxr1 | Prokr1 | Antxr1 | Lrig1 | **Gp9** | **Gp9** | Htra2 | Fbln2 | Bmp10 | Lrig1 | Pcyox1 |
| **Tacr1** | Htra2 | Antxr1 | Fbln2 | **Cd207** | Htra2 | Stambp | Antxr1 | **Tacr1** | **Gp9** | Loxl3 | Antxr1 | **Tacr1** | Prokr1 |
| Lrig1 | Klf15 | **Cd207** | Lrig1 | Mcm2 | Ruvbl1 | Loxl3 | Slc6a6 | Prokr1 | Prokr1 | Adamts9 | Anxa4 | Cntn3 | Rab11fip5 |
| **Dok1** | **Clec4f** | **Clec4f** | **Gp9** | Gpr27 | Pcyox1 | **Dok1** | Prokr1 | **Cd207** | **Dok1** | Stambp | Htra2 | Slc6a6 | **Tacr1** |
| Mcm2 | Fbln2 | Tgfa | **Cd207** | **Dok1** | Wnt7a | **Cd207** | Cntn3 | Antxr1 | Arhgap25 | Htra2 | **Gp9** | Hk2 | Prok2 |
| Tgfa | Pcyox1 | Pcyox1 | Tgfa | Stambp | **Cd207** | **Tacr1** | Tgfa | Lrig1 | Mcm2 | **Clec4f** | Pdzrn3 | Cnbp | Fbln2 |
| **Clec4f** | **Gp9** | Mcm2 | Loxl3 | V1ra8 | **Tacr1** | Prokr1 | Klf15 | Loxl3 | Arl6ip5 | Mcm2 | Pcyox1 | Antxr1 | Mcm2 |
| Htra2 | Antxr1 | **Tacr1** | Prokr1 | Htra2 | **Clec4f** | Antxr1 | Arl6ip5 | Sema4f | Rab11fip5 | Klf15 | **Cd207** | Dctn1 | Dguok |
| Pcyox1 | Sec61a1 | Lrig1 | **Tacr1** | Slc6a6 | Fbln2 | Hdac11 | Plxna1 | **Clec4f** | Stambp | **Tacr1** | Loxl3 | Fbln2 | Antxr1 |
| **Gp9** | Ruvbl1 | Slc6a6 | Arl6ip5 | Plxna1 | **Gp9** | Nagk | Tia1 | **Dok1** | Podxl2 | Snrpg | Actg2 | Anxa4 | Tgfa |
| Cntn3 | Cd207 | **Gata2** | Bmp10 | **Gp9** | Tgfa | Cntn3 | Pcyox1 | Slc6a6 | Sema4f | Fbxo41 | Arl6ip5 | Mcm2 | Loxl3 |
| Stambp | Zfml | Cntn3 | Mcm2 | Nup210 | Mcm2 | Tex261 | Anxa4 | Htra2 | Aak1 | Pcyox1 | Grip2 | Wnt7a | Mthfd2 |
| Tmf1 | Mthfd2 | Htra2 | Hdac11 | Antxr1 | Dys | Arhgap25 | Sec61a1 | Tgfa | Cntn3 | Dctn1 | Slc6a6 | Htra2 | Klf15 |
| Slc6a6 | Bmp10 | **Dok1** | Pcyox1 | **Clec4f** | **Dok1** | Tmf1 | Tmf1 | Arl6ip5 | Trh | Antxr1 | Cntn3 | Zfml | Suclg2 |
| Prokr1 | Aldh1l1 | Gpr27 | Wnt7a | Arhgap25 | Prokr1 | Htra2 | Bmp10 | Plxna1 | Fbln2 | H1fx | Mcm2 | Pcyox1 | Sec61a1 |

**B**

| **Lung** | **Respiratory**  **AND infection** | **Infection** | **GO: Immune**  **AND response** | **GO: cytokines** | **GO: chemotaxis** | |
| --- | --- | --- | --- | --- | --- | --- |
| Xpc | **Tacr1** | **Cd207** | Aplf | Tia1 | **Foxp1** | |
| **Dok1** |  | Lrig1 | **Tacr1** | Anxa4 | Sema4f | |
| Pas1c |  | **Tacr1** | **Foxp1** | Tmf1 | Prok2 | |
| **Tacr1** |  |  | **Gata2** | Mcm2 |  | |
| **Cd207** |  | | **Cd207** | **Dok1** |  |  |
|  |  |  | Tmf1 | Plxna1 |  |  |
|  |  |  | Ppp4r2 | **Tacr1** |  |  |
|  |  |  | Mgll | Wnt7A |  |  |
|  |  |  | Mitf | Bmp10 |  |  |
